# European public mental health responses to the COVID-19 pandemic

**DOI:** 10.1093/eurpub/ckac169

**Published:** 2022-11-21

**Authors:** Naomi Wilson, Shari McDaid, Frank Wieber, Jutta Lindert

**Affiliations:** The Mental Health Foundation, Glasgow, UK; The Mental Health Foundation, Glasgow, UK; Zurich University of Applied Sciences, Zurich, Switzerland; University of Applied Sciences Emden/Leer, Emden, Germany

## Abstract

**Background:**

The coronavirus disease 2019 (COVID-19) pandemic increased multiple risk factors for mental health. Evidence-based, intersectoral public mental health responses are therefore critical. The primary aim of this study was to collate public mental health responses from across Europe.

**Methods:**

We conducted a cross-sectional survey in March 2021. Participants were public and mental health professionals from across Europe. We developed an online instrument exploring five domains: changes in mental health supports during the pandemic; mental health support for vulnerable groups; multi-sectoral and service-user involvement; published mental health response plans; and perceived quality of overall country response.

**Results:**

Fifty-two individuals from 20 European nations responded. Reported changes in mental health supports included an increase in online mental health supports (*n* = 18); but no change in long-term mental health funding (*n* = 13); and a decrease in access to early interventions (*n* = 9). Responses indicated mental health support for vulnerable groups was limited, as was multi-sectoral and service-user involvement. Few national mental health response plans existed (*n* = 9) and 48% of respondents felt their countries mental health response had been ‘poor’ or ‘very poor’.

**Conclusions:**

Our results give insights into the changes in mental health support at a country level across Europe during the COVID-19 pandemic. They indicate countries were not prepared to respond and people with existing vulnerabilities were often neglected in response planning. To be prepared for future pandemics and environmental disasters Public Mental Health preparedness plans are highly needed. These must be developed cross-departmentally, and through the meaningful inclusion of vulnerable groups.

## Introduction

The coronavirus disease 2019 (COVID-19) pandemic has resulted in an increase in awareness of mental health conditions globally.^1^ The rapid spread of the coronavirus pandemic in 2019, resulted in unpredictability and uncertainty in the lives of millions.^2^ Simultaneously, the associated lockdown measures adopted by many countries resulted in periods of physical isolation, inactivity, and for some, loss of income and limited access to basic services.^2^ Consequently, a rise in the prevalence of those reporting mental health distress was observed in some studies,^3^ which healthcare and psychosocial systems in many European countries were not adequately equipped to respond to with epidemic measures.^4^ International organizations, including the World Health Organization, have since advocated for the integration of mental health supports into all countries pandemic response plans to mitigate these current difficulties and to prevent future public mental health crises.^5^

Public mental health was an area of acute and growing concern, even prior to the beginning of the COVID-19 pandemic.^1^ Specifically, several international documents and policies had recognized the need for upscaling public awareness of mental health across Europe and increasing primary prevention measures aimed at reducing the incidence of mental health difficulties.^6^ Despite this, in most countries, Public Mental health approaches remain inadequate.^7^

To design effective responses, a better understanding of the variations in public mental health responses to the pandemic from across Europe is necessary. Research exploring such responses is limited.^8^ However, while it is clear many responses possess common features, such as the expansion of digital mental health supports and mental health awareness campaigns, they also differ significantly in their content and focus.^9^ For example, at a national level, in the UK: while Wales chose to update their existing national mental health strategy; Northern Ireland incorporated a dedicated COVID-19 response within a wider mental health action plan; and both Scotland and England published separate response plans. Other European countries did not include mental health support in their COVID-19 response plan at all.^10^

The primary aim of this study is therefore to collate public mental health responses to the COVID-19 pandemic from across Europe. Specifically, we intended to explore five areas, namely: (i) changes in mental health supports; (ii) mental health support for high-risk groups; (iii) multi-sectoral and service-user involvement; (iv) published mental health response plans; and (v) perceived quality of overall response. Our secondary aim was to compare variations in these reported responses according to: (i) the cumulative number of COVID-19 cases per 100 000 of the population; and (ii) the gross domestic product (GDP) per capita, in the country respondents resided.

## Methods

### Design

A cross-sectional online survey was conducted in March 2021.

### Sampling and procedure

Purposive sampling was utilized to recruit public and mental health professionals from across Europe. This was done through an invitation email, which was sent directly to all members of the Public Mental Health Section of the European Association of Public Health (EUPHA), a network of researchers, policymakers and practitioners working in the field of public mental health, with one follow-up email sent 1 week later. In addition, the survey was sent directly to 21 public mental health professionals, in 9 different countries who were selected due to their level of knowledge and experience at a national level. They were also encouraged to distribute the survey to colleagues who they deemed to be experts in the field.

### Participants

Participants were members of the Public Mental Health Section of EUPHA and other public mental health professionals from across Europe. Inclusion criteria were working in the mental health field, within the European region during the pandemic. No exclusion criteria were applied.

### Instrument

The instrument consisted of four sociodemographic and professional items; and 10 public mental health items, covering 5 broad topics as outlined below (Supplementary appendix S1).

- Sociodemographic and professional itemsWe included the country where respondents worked, their professional role, the organization they worked for and their years of professional experience.
- Public mental health topics and items
  1. Perceived changes in available mental health supports
  2. Mental health supports for vulnerable groups
  3. Multi-sectoral approaches, service-user involvement and cross-departmental collaboration
  4. Published national, regional and local public mental health responses
  5. Mental health data collection and perceived quality of overall public mental health response

### Analysis

All quantitative data were collated and analyzed using SPSS v26. Data from each item were first analyzed for normal distribution using the Shapiro–Wilk test for normality.^11^ As responses to many of the items did not follow a normal distribution curve, non-parametric tests were utilized for all further analyses. Type I error rate was set to *α* = 0.05 and 95% confidence intervals were applied. Questions where the majority of respondents answered ‘don’t know’ were excluded from further analyses. Primary analyses were completed using the non-parametric Friedman test of differences and Chi-squared tests of association. In order to compare responses, the countries where participants worked were grouped according to whether they were deemed to have a ‘High’ (>8000); ‘Medium’ (6000–8000); or ‘Low’ (<6000) cumulative number of COVID-19 cases per 100 000 of the population at the time of data collection.^12^ In addition, responses were grouped according to whether participants worked in a country with a relatively higher GDP per capita (>$75 000); a relatively lower GDP per capita (<$43 000); or GDP per capita, which lay between these values ($43 000–$75 000).^13^ Spearman rank correlations and chi-squared analyses were then used to explore if significant differences in countries responses existed according to either of these groupings.

## Results

### Sociodemographic characteristics, professional characteristics and geographic spread

**Figure 1:**
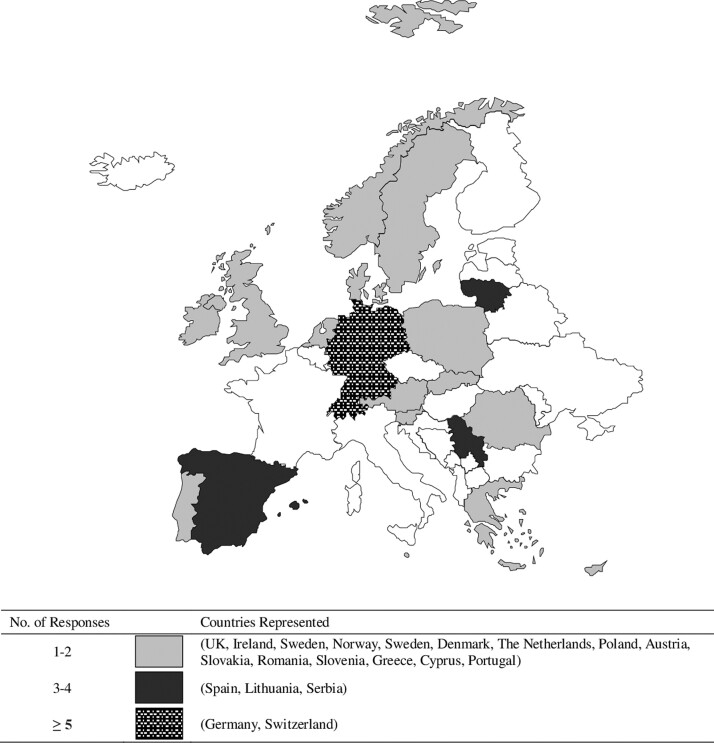
Geographical distribution of responses

Overall, we received 52 completed surveys all of which were included in our analyses (table 1). Responses were received from 20 European countries in total. The geographic spread of responses is shown in figure 1. The largest proportion of respondents reported working in a country deemed to have a ‘High’ number of COVID-19 cases (48%), and a GDP per capita of over $75 000 (38.5%) (table 1). The largest profiled groups of responses were from public health professionals (40%), working in a public health service (37%), who had over 20 years of professional experience (33%) (table 1).

**Table 1:** Sociodemographic and professional characteristics

Variable	*N*	(%)
Country’s cumulative COVID-19 cases per 100 000		
High (>8000) (Sweden, Slovenia, the Netherlands, Lithuania, Switzerland, Portugal and Serbia)	25	(48)
Medium (6000–8000) (Spain, Poland, Austria, Slovakia, Malta, Cyprus and UK)	12	(23)
Low (<6000) (Germany, Ireland, Romania, Denmark, Greece and Norway)	15	(29)
Country’s relative GDP per capita ($1000’s)		
High (>$75) (Switzerland and Ireland)	15	(29)
Medium ($43–$75) (Norway, Denmark, the Netherlands, Austria, Germany, Sweden, UK, Malta and Slovenia)	17	(33)
Low (<$43) (Spain, Serbia, Greece, Slovakia, Poland, Portugal, Romania, Lithuania and Cyprus)	20	(38)
Profession		
Public health professional	21	(40)
Mental health professional	9	(17)
Other health professional	4	(8)
Researcher	10	(19)
Policy professional	4	(8)
Other (civil servant/health service manager)	4	(8)
Years of professional experience		
0–5 years	11	(21)
6–10 years	9	(17)
10–15 years	7	(14)
15–20 years	8	(15)
>20 years	17	(33)
Organization		
Public health service	19	(37)
Other public sector agency	5	(9.6)
Academic institution	16	(30.8)
Non-governmental organization	7	(13)
Other	5	(9.6)

N = number of participants; % = percentage of participants.

## Public mental health topics

### Perceived changes in available mental health supports

**Figure 2:**
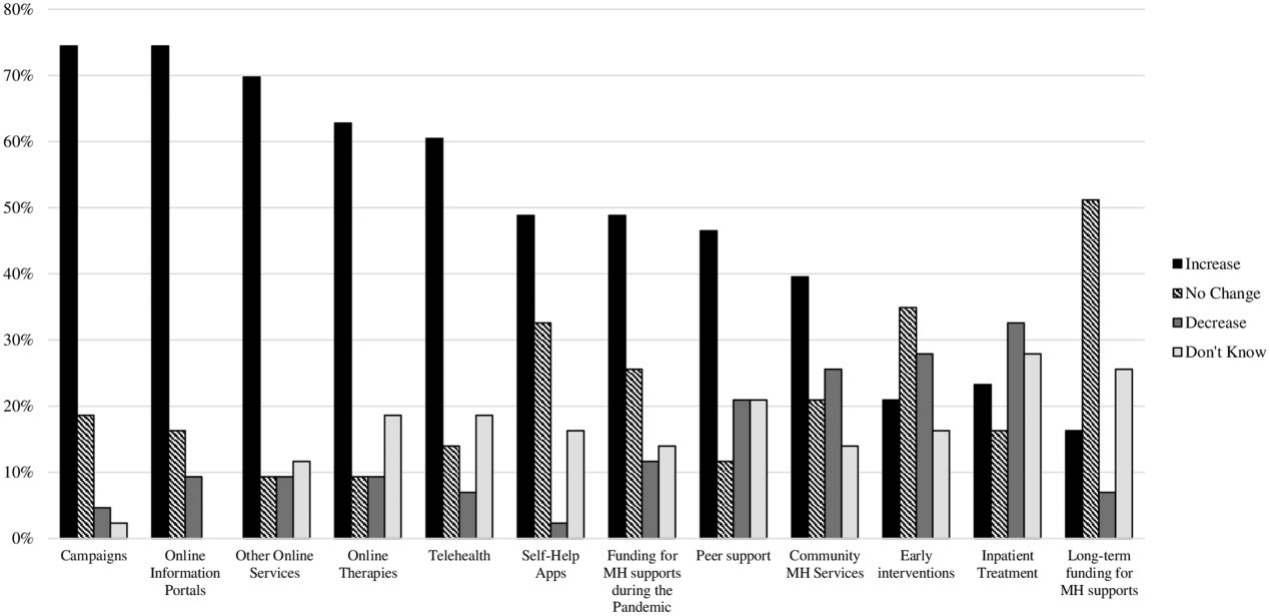
Perceived changes in available mental (MH) supports during the pandemic

Changes in the available mental health supports in the country where they worked during the pandemic was reported by the majority of respondents (87%, *n* = 45 respondents). Subsequent responses indicated that most of the forms of mental health support enquired about had increased, with the vast majority of respondents reporting an increase in mental health awareness campaigns (74%, *n* = 38 of respondents from 16 countries); online information portals (74%, *n* = 38 of respondents from 18 countries); online blended therapy (63%, *n* = 33 of respondents from 17 countries); telehealth (60%, *n* = 31 of respondents from 17 countries); and other forms of online services (70%, *n* = 36 of respondents from 16 countries). However, the largest proportion of respondents indicated that long-term funding for mental health supports had remained unchanged (51%, *n* = 27 of respondents from 13 countries) and that there had been a decrease in access to inpatient mental health services (40%, *n* = 21 of participants from 11 countries) and early interventions (42%, *n* = 22 of participants from 9 countries) (figure 2).

The changes made to the various forms of mental health support enquired about differed significantly, χ^2^ F (11) = 87.18, *P* < 0.0001. Whereas online blended therapy or counselling [median = 2.00 (interquartile range = 2.00)]; and Telehealth [2.00 (2.00)] increased; overall early interventions including primary care mental health services [−1.00 (3.00)] and inpatient mental health services [−1.00 (1.00)] decreased; and long-term funding for mental health supports did not change [0.00 (0.00)].

Spearman rank correlations did not show any significant association between changes in any of the forms of mental health support and either a country’s cumulative COVID-19 cases per 100 000 of the population, or their GDP per capita.

### Mental health support for vulnerable groups

Regarding the amount of attention given to various vulnerable sociodemographic groups, cross tabulations and chi-squared analysis of responses revealed that they significantly differed, χ^2^ = 83.12, df=20, *P*<0.0001. Compared to what would be expected if all groups had been considered equally, more participants reported that children (51.2%, *n* = 27), young people (51.2%, *n* = 27), older adults (81.1%, *n* = 42) and victims of domestic abuse or violence (50%, *n* = 26) had frequently or always been given specific attention whereas low-income families (38.9%, *n* = 20); people with a long-term physical health condition or disability (50%, *n* = 26); people with pre-existing mental health difficulty (42.9%, *n* = 22) and ethnic minorities (45.7%, *n* = 24) had rarely been given specific attention. Finally, respondents were significantly more likely to report those experiencing unemployment (42.9%, *n* = 22); homelessness (43.2%, *n* = 23) or who identified as LGBTQ+ (55.9%, *n* = 29) were never given specific attention for increased mental health support during the pandemic (figure 2).

Spearman rank correlations subsequently showed that the higher a country’s cumulative COVID-19 cases per 100 000 of the population, the more frequently respondents reported that mental health supports for low-income families (*r*_s_=0.49, *P* = 0.0057); older adults (*r*_s_ = 0.46, *P* = 0.0073); and individuals who identified as LGBTQ+ (*r*_s_ = 0.37, *P* = 0.04) had been considered. In addition, a country’s GDP per capita was positively associated with the frequency with which support for victims of domestic violence was considered (*r*_s_=0.343, *P* = 0.05).

### Multi-sectoral approaches, service-user involvement and cross-departmental collaboration

Regarding the sectors, which had been involved in coordinating their country’s response, participants’ reports also differed significantly, χ^2^ = 37.96, df = 16, *P* = 0.0015. Overall, no sector was reported as being involved by a majority of participants. Sectors that were most often reported to be ‘frequently’ involved were education (27%, *n* = 15) and social welfare (37%, *n* = 20), whereas sectors that were most often reported to be ‘never’ involved were housing (22%, *n* = 11) and criminal justice sectors (24%, *n* = 12) (table 2).

**Table 2:** Multi-sectoral involvement, cross-departmental collaboration and service user involvement in mental health responses to the pandemic

Questionnaire item	Respondents in agreement		Countries represented	
The following sectors were frequently involved in developing this response …	(*n*)	%	(*n*)	%
1. Education	(15)	27	(9)	45
2. Housing	(5)	10	(5)	25
3. Social welfare	(20)	37	(12)	60
4. Criminal justice	(5)	10	(4)	20
5. Immigration	(7)	14	(4)	20

**A structure for cross-departmental collaboration on mental health exists at a …**	**(*n*)**	**%**	**(*n*)**	**%**

1. National level	21	43	10	50
2. Regional level	17	35	6	30
3. Local level	14	29	6	30

**In developing this response those with lived experience of mental health difficulties were …**	**(*n*)**	**%**	**(*n*)**	**%**

1. Directly involved in the planning	1	2	1	5
2. On an advisory group	4	8	4	20
3. Consulted	6	12	5	25
4. Not involved at all	12	23	8	40
5. I do not know	29	56	11	55

More than 40% of participants reported that there was a structure for cross-departmental collaboration on mental health at a national level (43%, *n* = 21) (table 2). However, chi-squared analyses showed that respondents working in countries with a relatively lower GDP per capita were less likely to report such a structure existed (χ^2^ = 13.67, df = 6, *P*=0.03).

Finally, the majority of respondents also reported that they either did not know whether those with lived experience of mental illness had been involved in developing their country’s COVID-19 mental health response and recovery plan (56%, *n* = 29) or that they had not been involved at all (23%, *n* = 12) (table 2).

### Published national, regional and local public mental health responses to the pandemic

The largest proportion of respondents (29%, *n* = 15 respondents from 11 countries) reported that to date, no national COVID-19 public mental health response or recovery plan had been published in the country where they worked. Of the remainder, 25% (*n* = 13, from 9 countries) reported a published national plan did exist, 21% (*n* = 11, from 7 countries) reported this was in development and 25% (*n* = 13, from 8 countries) reported they did not know whether this existed or not. In addition, the largest proportion also reported that they did not know whether any regional (37%, *n* = 19 from 11 countries) or local (37%, *n* = 19 from 12 countries) COVID-19 public mental health response plans existed.

### Perceived quality of overall public mental health response

The majority of respondents (65.4%, *n* = 34) reported that data on the mental health effects of the pandemic in their country had been collected during the pandemic.

Finally, the highest proportion of participants reported that in their view their country’s response to the pandemic so far had either been ‘poor’ or ‘very poor’ (48.1%, *n* = 25). However, a large proportion also felt their country’s response to date had been ‘fair’ (42.31%, *n* = 22), while only 9.6% (five participants) reported the response was good. Spearman rank correlations subsequently showed that respondents working in wealthier countries rated their country’s mental health response higher.

## Discussion

The purpose of this survey was to collate public mental health responses to the COVID-19 pandemic from across Europe and to compare clustered groups of countries’ responses according to their cumulative number of COVID-19 cases at the time of data collection, as well as according to the GDP per capita in the countries referred to, in order to gather a picture of responses to date and inform any future adaptions. Overall, we collected the views of 52 individuals from 20 European countries. Their responses have highlighted some potential shortcomings in mental health responses to the pandemic, from which lessons can be learned.

Responses indicated that in order to deal with the consequences of the pandemic, countries largely moved to provide support online or remotely—a finding, which echoes those of other studies.^14^ Though some countries reportedly increased mental health funding, which can be assumed to support the shift to online services, others seemingly chose to increase online services while not increasing mental health funding. This is consistent with historical challenges for mental health budgets, which have traditionally struggled to achieve parity with those for physical health.

Participants also reported that the needs of many of those who are likely to have been disproportionally affected by the pandemic, i.e. those who were unemployed or living on a low-income, homeless, had a pre-existing physical or mental health condition, identify as LGBTQ+, or belong to an ethnic minority were rarely or never given specific consideration in their country’s mental health response planning. This is in keeping with other analyses of COVID-19 public mental health responses.^15^ Although being a country with higher COVID-19 case numbers and having higher GDP was associated with more support for vulnerable groups, this finding is of concern both because these social and cultural statuses generally carry higher risk of developing a mental health disorder and because these groups were affected by the pandemic in specific ways that seem to have gone unrecognized in response planning.

In many countries, several key sectors appear to have been lacking in the mental health response planning, and less than half of respondents reported a structure for cross-departmental collaboration on mental health at a national level existed—with low GDP countries being even less likely to have such structure. These findings are consistent with a recent WHO survey^10^ and run counter to Objective 6 of the European Mental Health Action Plan 2013–20. In addition, our results suggest a lack of visible service-user involvement in countries’ response planning, with nearly a quarter of respondents indicating that service users had not been involved in this process ‘at all’ while more than half stating that they did not know.

Only a quarter of participants reported that a national COVID-19 public mental health response or recovery plan had been published in their country. This suggests that despite a Europe-wide commitment to focus on and invest in population well-being (e.g. European Mental Health Action Plan 2013–20), in some countries’ government planning, mental health continues to lag behind physical health and efforts to prioritize it are still required—even in wealthier countries that reported a better quality of response.^16^

Finally, it emerged that several aspects of countries reported responses were positively correlated with their GDP. As such countries with a higher GDP not only tended to have better mental health services before the pandemic, but were also more able to increase their mental health funding in response. As such, although increased investment in public mental health is universally required, coordinated action is needed to ensure inequalities in mental health and mental healthcare across Europe are not widened further.

### Strengths and limitations

There are several limitations to our study. Firstly, our small number of respondents (*n* = 52) is a clear limitation. Moreover, we did not gather details on the age or gender of respondents, were unable to calculate a response rate and as we received only a few responses for each country included, we were unable to compare and contrast individual country-level responses, making generalizations difficult. In addition, as our sample population were recruited purposively, and some of this was done through membership of a professional organization, there is the potential for selection bias to have been introduced. The majority of respondents also worked in the health sector. This may account for the high number of ‘don’t know’ responses to questions related to other sectors and departments, which made several results difficult to interpret. Lastly, the cross-sectional nature of our survey limited our ability to explore adaptations in mental health services longitudinally over the course of the pandemic.

Despite this, our study has several strengths. Our survey was widely distributed, and we received responses from a diverse range of countries, allowing for a broad overview of responses from across Europe. In addition, questions centred on current or recent changes in mental health service provision, limiting the risk of recall bias; while the online and anonymous nature of the survey allowed for more accurate reporting amongst the sample population. Finally, the majority of those who participated appeared to have been highly experienced practitioners, potentially increasing the likelihood that they were aware of changes in mental health policy during the pandemic.

## Conclusions

Countries’ public mental health systems were not prepared to respond to the COVID -19 pandemic. As such, it has challenged societies and institutions. Our data suggest that while systems tried to adapt using digital healthcare, short-term funding for mental health was inconsistent, and long-term funding for mental health was mostly unchanged. As the mental health effects of the current and future pandemics are likely to be long-lasting and significant,^17^ we suggest increases in long-term funding for Public Mental health are urgently needed.

It also emerged that more than a year after the start of the pandemic, many countries had still not published a mental health response. To be prepared for coming challenges, such as other pandemics and environmental disasters, Public Mental Health preparedness plans are needed. These plans must be developed cross-departmentally and should incorporate the views of those with lived experience.

## Supplementary data

Supplementary data are available at *EURPUB* online.

*Conflicts of interest*: None declared.

## Supplementary Material

ckac169_Supplementary_Data

## Data Availability

The data underlying this article will be shared on reasonable request to the corresponding author. The COVID pandemic increased vulnerabilities, which existed before the pandemic. Face-to-face mental health services were interrupted, online mental health supports increased. Short-term increases in mental health funding were inconsistent, long-term funding remained unchanged. Service-user and multi-sectoral involvement was lacking in the pandemic responses. Nearly, a third of respondents reported no national public mental health response plan had been published.
