# Supplementary material for: European public mental health responses to the COVID-19 pandemic
Source: Eur J Public Health. 2022 Nov 21;33(1):87–92. doi: 10.1093/eurpub/ckac169 (PMC9897993; doi:10.1093/eurpub/ckac169)
Supplement: ckac169_Supplementary_Data [file ckac169_supplementary_data.docx]

Appendix 1. Online Survey

| **Sociodemographic Questions** | | | | | | | | | | | | | | | | | | | | | | | | | | | | | | | | | | | | | | | | | |
| --- | --- | --- | --- | --- | --- | --- | --- | --- | --- | --- | --- | --- | --- | --- | --- | --- | --- | --- | --- | --- | --- | --- | --- | --- | --- | --- | --- | --- | --- | --- | --- | --- | --- | --- | --- | --- | --- | --- | --- | --- | --- |
| **Q1. Please indicate the country within which you work** | | | | | | | | | | | | | | | | | | | | | | | | | | | | | | | | | | | | | | | | | |
| All European nations were listed as possible responses. | | | | | | | | | | | | | | | | | | | | | | | | | | | | | | | | | | | | | | | | | |
| **Q2. Please indicate your professional role** | | | | | | | | | | | | | | | | | | | | | | | | | | | | | | | | | | | | | | | | | |
| Public Health Professional | | Mental Health Professional | | | Other Health Professional | | | | Researcher | | | | Policy Professional | | | | | | Civil Servant | | | | | Health Service Manager | | | | Public Sector Manager | | | | | Student | | | | | | Other (Please specify) | | |
| **Q3. Please indicate your years of professional experience** | | | | | | | | | | | | | | | | | | | | | | | | | | | | | | | | | | | | | | | | | |
| 0-5 | | | | | 6-10 | | | | | | | | 10-15 | | | | | | | | | | | 15-20 | | | | | | | | | >20 | | | | | | | | |
| **Q4. Please indicate the type of organisation you work for** | | | | | | | | | | | | | | | | | | | | | | | | | | | | | | | | | | | | | | | | | |
| Public Health Service | | | Private Health Service | | | | | | | Public Sector Agency | | | | | | | | Academic Institution | | | | | | | | Non-Governmental Organisation | | | | | | | | | | Other | | | | | |
| **Public Mental Health Questions** | | | | | | | | | | | | | | | | | | | | | | | | | | | | | | | | | | | | | | | | | |
| **Topic 1: Perceived Changes in Available Mental Health Supports** | | | | | | | | | | | | | | | | | | | | | | | | | | | | | | | | | | | | | | | | | |
| **Q1. Are you aware of any changes in the mental health support in your country during the pandemic?** | | | | | | | | | | | | | | | | | | | | | | | | | | | | | | | | | | | | | | | | | |
| Yes | | | | | | | | | | | | | | | | | | | No | | | | | | | | | | | | | | | | | | | | | | |
| **Q2. How did the mental health support in your country, during periods of high social restriction in the pandemic, change in the following areas?** | | | | | | | | | | | | | | | | | | | | | | | | | | | | | | | | | | | | | | | | | |
| **(a) Mental Health Campaigns** | | | | | | Large Decrease | | | | | Medium Decrease | | | | | | Small Decrease | | | | | No Change | | | | | Small Increase | | | | | Medium Increase | | | | | | | | Large Increase | |
| **(b) Online Information Portals** | | | | | | Large Decrease | | | | | Medium Decrease | | | | | | Small Decrease | | | | | No Change | | | | | Small Increase | | | | | Medium Increase | | | | | | | | Large Increase | |
| **(c) Online Self-Help Apps** | | | | | | Large Decrease | | | | | Medium Decrease | | | | | | Small Decrease | | | | | No Change | | | | | Small Increase | | | | | Medium Increase | | | | | | | | Large Increase | |
| **(d) Online Therapies** | | | | | | Large Decrease | | | | | Medium Decrease | | | | | | Small Decrease | | | | | No Change | | | | | Small Increase | | | | | Medium Increase | | | | | | | | Large Increase | |
| **(e) Other Online Services** | | | | | | Large Decrease | | | | | Medium Decrease | | | | | | Small Decrease | | | | | No Change | | | | | Small Increase | | | | | Medium Increase | | | | | | | | Large Increase | |
| **(f) Telehealth** | | | | | | Large Decrease | | | | | Medium Decrease | | | | | | Small Decrease | | | | | No Change | | | | | Small Increase | | | | | Medium Increase | | | | | | | | Large Increase | |
| **(g) Social Prescribing** | | | | | | Large Decrease | | | | | Medium Decrease | | | | | | Small Decrease | | | | | No Change | | | | | Small Increase | | | | | Medium Increase | | | | | | | | Large Increase | |
| **(h) Early Interventions** | | | | | | Large Decrease | | | | | Medium Decrease | | | | | | Small Decrease | | | | | No Change | | | | | Small Increase | | | | | Medium Increase | | | | | | | | Large Increase | |
| **(i) Community Mental Health Services** | | | | | | Large Decrease | | | | | Medium Decrease | | | | | | Small Decrease | | | | | No Change | | | | | Small Increase | | | | | Medium Increase | | | | | | | | Large Increase | |
| **(j) Peer Support** | | | | | | Large Decrease | | | | | Medium Decrease | | | | | | Small Decrease | | | | | No Change | | | | | Small Increase | | | | | Medium Increase | | | | | | | | Large Increase | |
| **(k) Inpatient Treatment** | | | | | | Large Decrease | | | | | Medium Decrease | | | | | | Small Decrease | | | | | No Change | | | | | Small Increase | | | | | Medium Increase | | | | | | | | Large Increase | |
| **(l) Funding for mental health in the pandemic** | | | | | | Large Decrease | | | | | Medium Decrease | | | | | | Small Decrease | | | | | No Change | | | | | Small Increase | | | | | Medium Increase | | | | | | | | Large Increase | |
| **(m) Long-term funding for mental health** | | | | | | Large Decrease | | | | | Medium Decrease | | | | | | Small Decrease | | | | | No Change | | | | | Small Increase | | | | | Medium Increase | | | | | | | | Large Increase | |
| **Topic 2: Mental Health Support for Vulnerable Groups** | | | | | | | | | | | | | | | | | | | | | | | | | | | | | | | | | | | | | | | | | |
| **Q3. Please indicate which social groups have been given specific attention for mental health support during the pandemic.** | | | | | | | | | | | | | | | | | | | | | | | | | | | | | | | | | | | | | | | | | |
| **(a) Children** | | | | | | | | Never | | | | | | | Rarely | | | | | | Frequently | | | | | | | | | | Always | | | | | | | I don’t know | | | |
| **(b) Young Adults** | | | | | | | | Never | | | | | | | Rarely | | | | | | Frequently | | | | | | | | | | Always | | | | | | | I don’t know | | | |
| **(c) Low-income families** | | | | | | | | Never | | | | | | | Rarely | | | | | | Frequently | | | | | | | | | | Always | | | | | | | I don’t know | | | |
| **(d) Unemployed People** | | | | | | | | Never | | | | | | | Rarely | | | | | | Frequently | | | | | | | | | | Always | | | | | | | I don’t know | | | |
| **(e) Older People** | | | | | | | | Never | | | | | | | Rarely | | | | | | Frequently | | | | | | | | | | Always | | | | | | | I don’t know | | | |
| **(f) People with physical conditions or disabilities** | | | | | | | | Never | | | | | | | Rarely | | | | | | Frequently | | | | | | | | | | Always | | | | | | | I don’t know | | | |
| **(g) People with a pre-existing mental health difficulty** | | | | | | | | Never | | | | | | | Rarely | | | | | | Frequently | | | | | | | | | | Always | | | | | | | I don’t know | | | |
| **(h) Ethnic Minorities** | | | | | | | | Never | | | | | | | Rarely | | | | | | Frequently | | | | | | | | | | Always | | | | | | | I don’t know | | | |
| **(i) Victims of Domestic Violence** | | | | | | | | Never | | | | | | | Rarely | | | | | | Frequently | | | | | | | | | | Always | | | | | | | I don’t know | | | |
| **(j) LGBTQ+ People** | | | | | | | | Never | | | | | | | Rarely | | | | | | Frequently | | | | | | | | | | Always | | | | | | | I don’t know | | | |
| **(k) Homeless People** | | | | | | | | Never | | | | | | | Rarely | | | | | | Frequently | | | | | | | | | | Always | | | | | | | I don’t know | | | |
| **(l) Low-income families** | | | | | | | | Never | | | | | | | Rarely | | | | | | Frequently | | | | | | | | | | Always | | | | | | | I don’t know | | | |
| **(m) Unemployed People** | | | | | | | | Never | | | | | | | Rarely | | | | | | Frequently | | | | | | | | | | Always | | | | | | | I don’t know | | | |
| **Topic 3. Multisectoral Approaches; Cross-Departmental Collaboration and Service-User Involvement** | | | | | | | | | | | | | | | | | | | | | | | | | | | | | | | | | | | | | | | | | |
| **Q4.**  **Have the following sectors been involved in developing the mental health response to the pandemic?** | | | | | | | | | | | | | | | | | | | | | | | | | | | | | | | | | | | | | | | | | |
| **(a) Education** | | | | | | | | Never | | | | | | | Rarely | | | | | | Frequently | | | | | | | | | | Always | | | | | | | I don’t know | | | |
| **(b) Housing** | | | | | | | | Never | | | | | | | Rarely | | | | | | Frequently | | | | | | | | | | Always | | | | | | | I don’t know | | | |
| **(c) Social Welfare** | | | | | | | | Never | | | | | | | Rarely | | | | | | Frequently | | | | | | | | | | Always | | | | | | | I don’t know | | | |
| **(d) Criminal Justice** | | | | | | | | Never | | | | | | | Rarely | | | | | | Frequently | | | | | | | | | | Always | | | | | | | I don’t know | | | |
| **(e) Immigration** | | | | | | | | Never | | | | | | | Rarely | | | | | | Frequently | | | | | | | | | | Always | | | | | | | I don’t know | | | |
| **Q5. Is there a structure for cross-departmental or cross-agency collaboration on mental health at national/regional and/or local levels in your country?** | | | | | | | | | | | | | | | | | | | | | | | | | | | | | | | | | | | | | | | | | |
| **(a) National** | | | | | | | | | | | | | | | | | | | | | Yes | | | | | | | | | | No | | | | | | | I don’t know | | | |
| **(b) Regional** | | | | | | | | | | | | | | | | | | | | | Yes | | | | | | | | | | No | | | | | | | I don’t know | | | |
| **(c) Local** | | | | | | | | | | | | | | | | | | | | | Yes | | | | | | | | | | No | | | | | | | I don’t know | | | |
| **Q6.**  **To what extent have people with lived experience of a mental health problem been involved in developing COVID-19 mental health response plans in your country?** | | | | | | | | | | | | | | | | | | | | | | | | | | | | | | | | | | | | | | | | | |
| Not At All | | | | Consulted | | | | | | | | On an Advisory Group | | | | | | | | | | | Directly Involved in Planning | | | | | | | | | | | I don’t know | | | | | | | |
| **Topic 4. Published National, Regional and Local Public Mental Health Responses** | | | | | | | | | | | | | | | | | | | | | | | | | | | | | | | | | | | | | | | | | |
| **Q7. Have there been any published national, regional (county, federal state or similar) or local plans on mental health in response to the pandemic and recovery?** | | | | | | | | | | | | | | | | | | | | | | | | | | | | | | | | | | | | | | | | | |
| **(a) National** | | | | | | | | Yes | | | | | | | | No | | | | | | | | | In Development | | | | | | | | | | I don’t know | | | | | | |
| **(b) Regional** | | | | | | | | Yes | | | | | | | | No | | | | | | | | | In Development | | | | | | | | | | I don’t know | | | | | | |
| **(c) Local** | | | | | | | | Yes | | | | | | | | No | | | | | | | | | In Development | | | | | | | | | | I don’t know | | | | | | |
| **Q8a/b/c. Please indicate which of the following priorities have been reflected in this national/regional/local response (select all that apply)** | | | | | | | | | | | | | | | | | | | | | | | | | | | | | | | | | | | | | | | | | |
| Access to Services | Addressing Inequalities | | | Mental Health Promotion/Prevention | | | Integrating Mental and Physical Healthcare | | | | | Digital/Online Innovation | | | | | | Rehabilitation and Recovery | | | | | Service User/Carer Involvement | | | | | | Workforce | | | | | Funding | | | | | | | Other (please specify) |
| **Topic 5. Mental Health Data Collection and Perceived Quality of Overall Public Mental Health Response** | | | | | | | | | | | | | | | | | | | | | | | | | | | | | | | | | | | | | | | | | |
| **Q9. Has there been public health data collection on the mental health effects of the pandemic in your country?** | | | | | | | | | | | | | | | | | | | | | | | | | | | | | | | | | | | | | | | | | |
| Yes | | | | | | | | | | No | | | | | | | | | | | | | | | | I don’t know | | | | | | | | | | | | | | | |
| **Q10. How would you rate the overall quality of . . .** | | | | | | | | | | | | | | | | | | | | | | | | | | | | | | | | | | | | | | | | | |
| **(a) your country’s response to the mental health effects of the pandemic so far?** | | | | | | Very poor | | | | | | | | Poor | | | | | | Fair | | | | | | | | | | Good | | | | | | | Excellent | | | | |
